# Health Care Professionals’ Attitudes Toward, and Experiences of Using, a Culture-Sensitive Smartphone App for Women with Gestational Diabetes Mellitus: Qualitative Study

**DOI:** 10.2196/mhealth.9686

**Published:** 2018-05-14

**Authors:** Lisa Garnweidner-Holme, Therese Hoel Andersen, Mari Wastvedt Sando, Josef Noll, Mirjam Lukasse

**Affiliations:** ^1^ Institute for Nursing and Health Promotion Faculty of Health Sciences Oslo Metropolitan University Oslo Norway; ^2^ Department of Technology Systems University of Oslo Oslo Norway

**Keywords:** mHealth, gestational diabetes mellitus, antenatal care, culture sensitivity

## Abstract

**Background:**

The increasing prevalence of gestational diabetes mellitus (GDM) among women of different ethnic backgrounds provides new challenges for health care professionals, who often find it difficult to provide information about the management of this disease to such individuals. Mobile health (mHealth) may act as a useful tool for blood sugar control and care process enhancement. However, little is known about health care professionals’ experiences and attitudes toward the use of mHealth for women with GDM.

**Objective:**

The aim of this study was to explore how health care professionals perceived the provision of care to pregnant women who managed their GDM using the culture-sensitive Pregnant+ app in a randomized controlled trial.

**Methods:**

Individual interviews with 9 health care professionals providing care for women with GDM were conducted. Braun and Clark’s method of thematic content analysis inspired the analysis. This study included health care professionals who were primarily responsible for providing care to participants with GDM in the Pregnant+ randomized controlled trial at 5 diabetes outpatient clinics in Oslo, Norway.

**Results:**

Health care professionals perceived mHealth, particularly the Pregnant+ app, as an appropriate tool for the care of women with GDM, who were described as individuals comprising a heterogeneous, motivated group that could be easily approached with health-related information. Some participants reported challenges with respect to provision of advice to women with different food cultures. The advantages of the Pregnant+ app included provision of information that women could access at home, the information provided being perceived as trustworthy by health care professionals, the culture sensitivity of the app, and the convenience for women to register blood sugar levels. Technical problems, particularly those associated with the automatic transfer of blood glucose measurements, were identified as the main barrier to the use of the Pregnant+ app. Strict inclusion criteria and the inclusion of participants who could not speak Norwegian were the main challenges in the recruitment process for the randomized controlled trial.

**Conclusions:**

The findings of this study suggest that mHealth is a useful tool to enhance the care provided by health care professionals to women with GDM. Future mobile apps for the management of GDM should be developed by a trustworthy source and in cooperation with health care professionals. They should also be culture sensitive and should not exhibit technical problems.

## Introduction

Gestational diabetes mellitus (GDM) is defined as glucose intolerance recognized for the first time during pregnancy [1]. The development of GDM may lead to short- and long-term health consequences for the mother and the newborn child [2,3]. The risk factors for GDM include advanced maternal age, maternal obesity, family history of diabetes, ethnicity, polycystic ovarian syndrome, and history of GDM [3,4].

The prevalence of GDM is increasing globally, and the rates may range from 1% to 20% depending on the screening procedures employed and the characteristics of the population [5-7]. In Norway, the prevalence of GDM is approximately 3.8% [8], although a cohort study in a suburb of Oslo reported an overall prevalence of 13%. Moreover, the prevalence rates were 14.6% among women of non-European origin and 11% among ethnic Norwegians [9]. Comparison of these findings with the national statistics suggests that the number of pregnant women with GDM in the general population is underestimated.

The increasing prevalence of GDM provides new challenges for health care professionals in antenatal care. In Norway, the team responsible for the care of women with GDM includes obstetricians, internists, midwives, and nurses with a specialization in diabetes care. At the time of this study, women with 75 g oral glucose tolerance test (OGTT) values of ≥9.0 mmol/L (2 h plasma glucose) received specialized care at diabetes outpatient clinics (DOCs) [10]. However, the new guidelines for GDM now recommend that only women with OGTT values of ≥11.0 mmol/L should receive care at DOCs [11], whereas those with values ranging between 9.0 mmol/L and 11.0 mmol/L should be followed up by primary health care professionals. The implication of this is that more health care professionals without specialized training in diabetes management will now be involved in the care of women with GDM.

The first-line treatment for GDM includes provision of information on the advantages of a healthy diet, physical activity, and regular blood sugar level measurements [12,13]. Previous research has shown that pregnant women, particularly those with GDM, can be easily approached with health-related information [14-16]. However, our previous study [Borgen, to be submitted for publication] focusing on a multi-ethnic population of pregnant women revealed poor levels of knowledge about GDM among this population at the time of first consultation. Moreover, non-native Norwegian speakers exhibited significantly poorer levels of knowledge about GDM compared with native Norwegian speakers. It has been previously shown that knowledge regarding the possible consequences of a disease acts as a motivator of behavioral change [17]. Therefore, it is of utmost importance that health care professionals provide women with sufficient information on the importance of controlling their blood sugar levels and the possible consequences in terms of future health.

Women diagnosed with GDM emphasize the need for individually tailored, culturally appropriate information [18]. The current care plan for women with GDM in Norway includes teaching them how to record their own blood glucose levels and providing them with verbal information on health and nutrition with or without accompanying it with written information. However, health care professionals find it challenging to communicate information about diet and physical activity, particularly when faced with a multicultural and socially diverse population [19,20]. There is general agreement that health promotion efforts in multicultural societies must be culture sensitive [21,22], and mobile health (mHealth) technologies can potentially serve as a new tool for the management of disease and promotion of healthy behavior [23]. Previous studies have shown that mHealth interventions may help patients control their blood sugar levels [24-26] and elicit patient engagement [27]. However, although mHealth can offer tailored information for different groups of individuals [28], there is still limited evidence on its effectiveness with regard to culture-sensitive interventions, one of the main challenges of which is the recruitment of participants with limited language skills [29]. The acceptance of mHealth among health care professionals plays a key role in the success of mHealth interventions, and two systematic reviews examining this previously reported divergent results [30,31]. White et al [30] concluded that health care professionals exhibited high levels of acceptance of mHealth, whereas the other systematic reviews on health care professionals’ acceptance of mHealth reported varied results based on different studies [31]. Little is known about the health care professionals’ experiences and attitudes toward culture-sensitive mHealth interventions for the management of GDM in antenatal care.

This study was part of the Pregnant+ randomized controlled trial (RCT) which tested the addition of a culture-sensitive mobile phone app to the standard care protocol for GDM and compared the findings to those of the standard care protocol alone in five different DOCs in Norway [32]. A total of 238 women were included in the study, of which 108 were native Norwegian speakers and 130 were non-native Norwegian speakers. The inclusion criteria were as follows: <33 weeks pregnant, a 2 h OGTT value of ≥9 mmol/L, age above 18 years, owning a mobile phone (iPhone or Android), and understanding Norwegian, Urdu, or Somali. The aim of the RCT was to determine whether use of the app resulted in better blood glucose values (measured using an oral glucose test 3 months postpartum) in women with GDM [32]. Nearly 40.0% of the non-native Norwegian speakers were Asian (39.5%), 22.5% were African, and 15.5 % were from Eastern Europe [Borgen, January 2018, manuscript submitted for publication]. The mobile phone app analyzed in this study supported automatic transfer of blood glucose values from the measurement device and provided a graphic overview of blood glucose values over time. Moreover, the Pregnant+ app included aspects of the surface structure of culture sensitivity, as defined by Resnicow et al [33]. This dimension of culture sensitivity included the use of pictures of people and food familiar to and preferred by the target population [21]. In addition, the app was available in Norwegian, Urdu, and Somali [34]. To tailor the contents of the app, the users could create their own profiles by providing the following information at the time of first login: (1) the outpatient clinic where they received health care and the hospital where they would give birth; (2) their perceived level of physical activity before pregnancy; (3) their preferred food culture; and (4) their weight and height before pregnancy. Pictures illustrating healthy eating varied in accordance with the patient’s chosen food culture, and emoticons were used to overcome language barriers. Patients with high blood sugar levels were immediately sent information on healthy eating and ways to control the levels. Patients could also register the amount of time spent in performing physical activities and were provided feedback if they met the recommendations of the national health authorities [24]. They also received information about physical activity that was tailored for their levels before pregnancy. Health care professionals involved in the care of participants were asked to refrain from using the Pregnant+ app as a communication tool in their consultation to avoid confounding.

The aim of this RCT targeting a multi-ethnic study population was to explore the attitudes and experiences of health care professionals using a culture-sensitive mobile phone app to manage GDM. In addition, the health care professionals’ general experiences with regard to provision of care to women diagnosed with GDM were also analyzed and described because previous evidence on this topic was very limited.

## Methods

This study included individual interviews with 9 health care professionals who provided care to pregnant women with GDM participating in the Pregnant+ RCT. A qualitative study design was chosen because mHealth interventions are considered to be complex to evaluate due to their novelty and different outcome measurements [35,36]. Moreover, the study design provided insights into the personal experiences of health care professionals [37].

### Interviews

The interviews were conducted by the second and third authors at the working sites of the participants between May and June 2017 and lasted for approximately 16–35 min. A semistructured interview format was pilot tested with one of the participants, and the main themes in the interview guide were (1) general experiences of providing care to women with GDM; (2) attitudes toward the use of mHealth; (3) experiences of recruiting participants for the Pregnant+ RCT; (4) and experiences of providing care to participants in the Pregnant+ RCT.

### Selection of Participants and Recruitment

The researchers aimed to interview all health care professionals responsible for recruitment and/or provision of care to participants in the Pregnant+ RCT. In total, the interviewers asked 11 health care professionals to participate in the study, of which one refused because he or she believed that they did not have much to contribute and another declined as he or she was unavailable. The 9 health care professionals who were willing to participate received verbal and written information about the study, and the study protocol was approved by the Norwegian Social Science Data Services (ID number: 2014/38942).

### Analysis

The interviews were audiotaped and transcribed by the second and third authors. The transcripts were read by the first author and randomly compared with the audiotapes to ensure accuracy of the transcription process. Braun and Clark’s method of thematic content analysis inspired the analysis [37] and included the following steps: (1) repeated reading of each informant’s transcripts to become familiar with the data; (2) generating initial codes (words or short phrases in the transcripts) relevant to the research questions; (3) organizing the codes into sub-themes; (4) arranging the sub-themes into overarching themes; and (5) defining and naming the themes. The second and third authors conducted the analysis and discussed potential codes and themes with the other authors.

## Results

### Characteristics of Study Participants

Table 1 shows the educational backgrounds and work experiences of the participants. One participant was a medical secretary who was involved in the recruitment process for the RCT but did not take part in patient care. A majority of the participants comprised midwives (n=6), and the remaining were nurses specialized in diabetes care (n=3). All the participants worked at DOCs, with a high population of women from different ethnic backgrounds. The participants’ involvement in the care of women with GDM depended on the working site because the organization of care varied with the DOCs. For instance, midwives were not responsible for the management of blood glucose levels at one DOC.

Data analysis identified three themes representing the participants’ attitudes toward and experiences of caring for pregnant women with GDM participating in the Pregnant+ RCT, and these were as follows: (1) general experiences of caring for women with GDM depicted the health care professionals’ motivation and perceived challenges toward caring for women with different ethnic backgrounds; (2) attitudes toward and experiences of using mHealth illustrated their personal attitudes toward mHealth tools and their previous experiences of using them for disease management and patient-client communication; and (3) experiences of using the Pregnant+ mobile phone app in the follow-up of women with GDM revealed the health care professionals’ evaluation of the Pregnant+ app, the facilitators and challenges of providing care to participants who had access to the Pregnant+ mobile phone app, and the professionals’ experiences of the recruitment process.

**Table 1:** Educational backgrounds and work experiences of the study participants. Fictional names have been used.

Participant	Educational background	Work experience
Kari	Midwife	Nurse for 6 years Midwife for 10 years 7 years at a DOC
Anne	Midwife	20 years at a DOC Specialization in diabetes care
Kristin	Midwife	Midwife for 20 years 5 years at a DOC
Nina	Midwife	Midwife for 10 years 5 years at a DOC
Linn	Diabetes specialist nurse	7 years at a DOC
Anette	Diabetes specialist nurse	Diabetes specialist nurse for 16 years 14 years at a DOC
Gunn	Diabetes specialist nurse	10 years at a DOC
Lise	Midwife	16 years at a DOC
Julie	Midwife	Midwife for 15 years 8 years at a DOC

### General Experiences of Caring for Women with Gestational Diabetes Mellitus

This overarching theme included 3 sub-themes: (1) motivation to provide care to women with GDM; (2) description of the characteristics of women with GDM; and (3) experiences of providing information about diet and physical activity.

The majority of the participants reported that they were strongly motivated professionally to provide continuous care to women with GDM. The participants described the women with GDM as being a very heterogeneous group with regard to their ethnic and socioeconomic backgrounds and were surprised to meet women who had developed GDM despite not exhibiting any of the known risk factors. In general, pregnant women with GDM were perceived as being very motivated and easy to approach with health-related information. This was illustrated by the following statement made by a participant who described the reaction of women after being diagnosed with GDM:

Women take it very seriously. Some get very sad. It happens very rarely that they don’t care.

Lise

The participants reported that it was very important for them to provide women with information about healthy eating and physical activity, especially because they found that the women appeared to have little knowledge about GDM. They felt that the majority of the women followed their advice, and it was very important for them to achieve long-term changes in the women’s health behaviors. All of the participants focused on the prevention of diet-related diseases in their consultations, as illustrated by the following statement by a midwife:

There should be more focus on preventing disease instead of treatment.

Nina

One midwife felt that it was important to build a good relationship with the women to achieve behavioral changes. However, the participants also reported experiencing challenges in providing dietary advice, mainly because the pregnant women were often confused by contradictory dietary information obtained from different health care professionals or the media. The participants also reported that women with GDM were often advised to adopt a low-carb diet or to stay away from all foods containing sugar.

All of the participants had experienced providing dietary advice to women with different ethnic backgrounds, and a majority of them did not find it difficult to adjust their advice to other food cultures. In fact, they emphasized that it was important for them to have this knowledge about different food cultures. However, two midwives reported finding provision of dietary advice to women with different food cultures challenging, and one statement made by a midwife suggested that she believed ethnic Norwegian women had more knowledge about diet than immigrant women:

Ethnic Norwegian women do often know what they have to do, but struggle to accomplish it; whereas immigrant women often get surprised about what they should do.

Nina

Another midwife reported challenges related to non-verbal communication with non-ethnic Norwegian women. For instance, she was unsure if women from South Asia understood what she told them because they were less expressive in their communication and provided fewer responses than ethnic Norwegian women. Several participants also experienced difficulties with consultations that included an interpreter and felt that pregnant women who needed an interpreter did not receive equal care. One midwife stated that they were unable to prioritize patients requiring an interpreter due to their busy schedule.

### Experiences and Attitudes Toward mHealth

The following sub-themes were identified within this theme: (1) former experiences with mHealth and (2) attitudes toward mHealth. All of the three nurses specialized in diabetes care and one midwife had previously experienced using mHealth in their consultations. The participants stated that a mobile app could be a useful tool during consultations, and one nurse specialized in diabetes care stated the following:

I use an app to provide information about carbohydrates. That’s useful since you always have it with you, because these leaflets always get lost.

Linn

However, two participants expressed barriers associated with using mobile apps during consultations with women with GDM. One participant felt that the app would not let a pregnant woman communicate all the emotions she was feeling adequately upon being diagnosed with GDM, and this would affect the participant’s communication/relationship with the woman.

Although half of the participants did not use mHealth apps personally, participants who had no previous experiences with mHealth mentioned several advantages of using mHealth during consultations. For instance, one midwife stated the following:

We have this information material that we show to the women, but I think this could get too much in the first consultation and I experience that I have to repeat things several times. So I think it would be good to have an app you can read undisturbed.

Kari

Other perceived advantages included those related to the management of GDM by women. Participants assumed that it would be more convenient for women to register their blood sugar values on a mobile phone compared with a booklet because the latter could be easily lost. They also thought that the use of mHealth would increase in the future and felt that it was important for them to keep up with new developments.

### Experiences with the Pregnant+ Mobile Phone App for the Management of Women with Gestational Diabetes Mellitus

Four sub-themes were identified in this theme: (1) professionals’ evaluation of the Pregnant+ app; (2) experiences with the Pregnant+ app in the care of women with GDM; (3) experiences with recruitment of participants for the RCT; and (4) organizational challenges. Eight of the participants had prior knowledge about the contents and features of the Pregnant+ app and felt that it had several advantages with regard to the follow-up of these women as well as their ability to manage their own GDM. For instance, health care professionals liked that the app contained a lot of different information that women could access repeatedly at any time after the consultation. Several participants mentioned that they had confidence in the contents of the Pregnant+ app and that the information was in agreement with their advice. They sometimes meet women with apps to manage their diabetes that were unknown to them and expressed that it could be difficult to know if they could rely or would approve of the content of these apps.

Although the health care professionals were asked to refrain from using the Pregnant+ app as an active communication tool during their consultations, their experiences of providing care to women who had access to it were recorded. Several participants had asked the pregnant women about their experiences with the Pregnant+ app and found that they preferred registering their blood sugar levels in the app and liked how the information was presented. On the other hand, the participants also encountered women who had experienced technical difficulties with the app, particularly with regard to the automatic transfer of blood sugar values from the measuring device to the mobile phone. The participants believed that technical issues could be a major barrier to the use of mHealth, both for self-management of GDM as well as during consultations.

Moreover, the participants stated that the Pregnant+ app could be a very useful tool for women with different backgrounds, mainly because it used simple and culture-sensitive illustrations that made the text more understandable. Although none of the participants could report experiences of using the Somali or Urdu versions of the app, they believed that English would be the most important language to reach women with different ethnic backgrounds. One participant was surprised that women from Somalian or Urdu ethnic backgrounds did not use the app in their own mother tongues. She related this to her experience of recruiting study participants for the RCT where it was difficult to include participants who could not speak Norwegian:

I was surprised that there were not more women who used the Somali or Urdu version of the app. It seems that those women who wanted to participate in the study, have good knowledge of the Norwegian language.

Anne

Although the participants felt that they received sufficient help from the research team, they found the recruitment process for the RCT challenging due to its strict inclusion criteria. Others struggled with the organization of care for women with GDM at their hospitals, and some participants stated that the lack of cooperation between the different health care professionals involved in the care process was a barrier to the recruitment process as well as the use of mHealth.

## Discussion

### Principal Findings

The results of this study showed that health care professionals perceived mHealth, particularly the Pregnant+ app, as an appropriate tool for the care and follow-up of women with GDM, who were described as individuals comprising a very heterogeneous and motivated group that could be easily approached with health-related information. Some participants reported challenges associated with providing advice to women with different food cultures. The advantages of the Pregnant+ app were provision of information that women could access at home, the provided information being trustworthy, the culture sensitivity of the app, and the convenience of automatic transfer of blood sugar levels to the mobile phones. Technical problems were mentioned as the main barrier to the use of the Pregnant+ app, whereas the strict criteria and inclusion of participants who could not speak Norwegian were the main challenges in the recruitment process.

There is growing evidence in support of the impact of mHealth interventions on the management of diabetes [26,38]. However, given that there is as large variety of different mHealth tools currently available, the most effective method and setting for the management of GDM remain unclear [39]. A previous systematic review concluded that there was insufficient evidence showing that the use of telemedicine technology was superior to the use of the standard care procedure for women with GDM [39]. Riga et al [40] reported promising results with regard to the acceptance of mobile phone telemedicine among pregnant women with GDM. However, little is known about the use of mobile phone apps for the management of GDM [25,41]. Health care professionals play an important role in the implementation and effectiveness of mHealth tools, irrespective of their type [31]. The findings of this study showed that health care professionals exhibited positive attitudes toward the use of mobile phone apps in the care of women with GDM. Previous studies examining health care professionals’ acceptance of mHealth have reported varying results [31], thus illustrating the need to examine the perceived advantages and disadvantages of using these tools in different settings. Although the participants exhibited positive attitudes toward the Pregnant+ app, some reported experiencing challenges in the recruitment of women for the RCT due to the strict inclusion criteria. Women with gluten or lactose tolerance were excluded from the RCT due to the lack of specific nutritional guidance for these women. To measure the effect of the app during pregnancy, women had to be able to have the app for at least 1 month before the second measurement point at 36 weeks of pregnancy; thus, women could not be included after 33 weeks of pregnancy. Twin pregnancies were excluded because the mode of delivery might have influenced the secondary outcomes might be influenced as mode of delivery. In addition, the app was only available in three languages due to the lack of resources necessary for translation.

One of the key advantages of mHealth, particularly the Pregnant+ app, was the possibility of providing vast quantities of health-related information that women could access repeatedly at their own convenience after the consultation. The participants in our study, who were strongly engaged professionally in the care of women with GDM, often felt that the women had difficulty remembering all of the information given to them. This was in agreement with other studies that had reported an information overload among pregnant women [16,42]. Similarly, another pilot test of an app that monitored gestational weight gain reported that it could help pregnant women cope with the great amounts of information provided to them by different sources [43]. Moreover, given the rapid increase in apps available for the management of GDM [44], the participants in our study stated that it was important that they could trust the developer and the information provided by the app. This finding was in agreement with another qualitative study examining pregnant women in Norway, where health care professionals were considered to be the most trustworthy source of health-related information [16].

Similar to previous reports, some participants in our study experienced difficulties providing diet-related information to women with different ethnic backgrounds [19] and stated that the Pregnant+ app could facilitate care for such women. There is a general agreement that efforts made to promote health need to be culture sensitive [45]. As described previously, the Pregnant+ app included features of the surface structure of culture sensitivity, as defined by Resnicow [33]. Participants in our study liked that the illustrations of diet-related information were adjusted for different food cultures. Another surface structure was the translation of health material into other languages, and many participants had previously experienced challenges with providing care with the help of an interpreter. Therefore, they appreciated that they could provide the women with the Pregnant+ app in their mother tongues. Although mHealth can offer tailored information for different groups of individuals [28], there is still limited evidence on the effectiveness of culture-sensitive mHealth interventions. For instance, an evaluation of meditation mobile phone apps that were culturally tailored found that this approach may be unnecessary [46]. As previously mentioned, one of the main challenges faced during the recruitment of women was limited language skills necessary for culture-sensitive mHealth interventions. Although the Pregnant+ app was available in Somali and Urdu, HCPs had difficulties in recruiting women who only understood these languages. Lopez-Class [29] previously developed strategies for the recruitment of immigrant participants, and the most relevant of these was customization of incentives for specific ethnicities and involvement of local community organizations relevant to immigrants.

To increase the effectiveness of mHealth interventions, possible disadvantages have to be overcome. Several participants reported technical issues as being the main barrier to using mHealth and the Pregnant+ app. The participants emphasized the convenience of automatic transfer of blood sugar levels as one of the most important advantages of the Pregnant+ app. However, this was also the feature that exhibited the most technical problems, and these challenges were mainly linked to software updates, either of the protocol being used to send the data from the glucometer to the mobile phone or of the operating system of the phone itself. A standard Bluetooth interface was used during the test and verification stage, while in the clinical intervention the meters had changed using Bluetooth low energy. As a result, not all phones used by the study participants were able to use Bluetooth low energy, and thus were hampered by a more cumbersome set-up and data exchange. This kind of incompatibility between older and newer versions of equipment is imminent in digital studies and cannot be avoided. However, sensor communication has developed considerably, and more than 97% of phones in countries like Norway are currently equipped to easily exchange data with sensors such as glucometers, suggesting that these problems are avoidable in the future. A recent review of health care professionals’ acceptance of eHealth also reported technical problems as a key limitation of mHealth tools [30]. In addition, one participant in our study was afraid that mHealth could interfere with her personal communication with the patient during their consultations, although there is some evidence that the use of mHealth tools can improve communication between health workers and their patients [47]. In the Pregnant+ study, health care professionals were asked to refrain from using the Pregnant+ app as a communication tool in their consultation to reduce possible confounding. Previous studies have reported that successful development and implementation of mHealth interventions should involve both the health care professionals and their clients [36,48]. For instance, a narrative review of mHealth technologies in the prevention and management of type 2 diabetes mellitus found that such technologies, with added support from the health care professionals, resulted in better outcomes for patients with type 2 diabetes mellitus than interventions that did not involve health care professionals [48].

### Limitations

The first limitation of this study was the small sample size, which is typical for qualitative studies [37]. However, the majority of the health care professionals involved in the Pregnant+ study participated in this study. Moreover, this study design was chosen as it could contribute to the complex evaluation of mHealth interventions [35]. Interviews were conducted among health care professionals who were involved in the recruitment process of the Pregnant+ study. Thus, experiences from other health care professionals such as the women’s general practitioners or internists were not included in this study, and these may affect the results. The health care professionals’ attitudes toward the app may be influenced by their knowledge of its contents. The participants of this study exhibited good knowledge of the contents of the app, and this must be taken into consideration when comparing the results of this study with those of previous studies examining the attitudes of health care professionals toward mHealth. Moreover, our findings suggest that the Pregnant+ app could be a useful tool for the improvement of communication between health care professionals and women of different ethnic backgrounds. However, this study was limited owing to the difficulties associated with recruitment of women who did not understand Norwegian, and further research targeting immigrant women is necessary.

### Comparison with Prior Work

To the best of our knowledge, this is the first study to investigate the experiences of health care professionals providing care for women managing GDM using a mobile phone app. The findings of this study showed high levels of acceptance of mHealth among the participants, and this was in agreement with a previous study [31]. Technical problems were identified as the main barrier to the use of mHealth in the care of patients [30,31]. This study also investigated the health care professionals’ experiences with immigrant women, although there is still limited evidence on the effectiveness of culture-sensitive mHealth interventions. Contrary to another study investigating the effectiveness of a culture-sensitive mobile phone app [46], the participants of the current study stated that the Pregnant+ app could be an appropriate tool for the care of immigrant women. Moreover, in accordance with a previous study [49], the participants reported difficulties associated with the recruitment of women from different ethnic backgrounds who did not speak the local language.

### Conclusions

The findings of this study suggest that mHealth acts as a useful tool to enhance the health care professionals’ experience of caring for women with GDM. Future mobile apps for the management of GDM should be developed by a trustworthy source and in cooperation with health care professionals. Moreover, efforts should be made to ensure that they are culture sensitive and do not exhibit technical problems. Further research targeting immigrant women who do not speak the local language are needed to determine the effects of culture-sensitive mHealth interventions.

## Abbreviations

DOC: diabetes outpatient clinic
GDM: gestational diabetes mellitus
RCT: randomized controlled trial
